# Reply to Assari and Lantz: Heterogeneity in BFY impacts

**DOI:** 10.1073/pnas.2201864119

**Published:** 2022-08-08

**Authors:** Sonya V. Troller-Renfree, Molly A. Costanzo, Greg J. Duncan, Katherine Magnuson, Lisa A. Gennetian, Hirokazu Yoshikawa, Sarah Halpern-Meekin, Nathan A. Fox, Kimberly G. Noble

**Affiliations:** ^a^Department of Biobehavioral Sciences, Teachers College, Columbia University, New York, NY 10012;; ^b^Institute for Research on Poverty, University of Wisconsin–Madison, Madison, WI 53706;; ^c^School of Education, University of California, Irvine, CA 92697;; ^d^Sandra Rosenbaum School of Social Work, University of Wisconsin–Madison, Madison, WI 53706;; ^e^Sanford School of Public Policy, Duke University, Durham, NC 27708;; ^f^Department of Applied Psychology, New York University, New York, NY 10012;; ^g^School of Human Ecology, University of Wisconsin–Madison, Madison, WI 53706;; ^h^LaFollette School of Public Affairs, University of Wisconsin–Madison, Madison, WI 53706;; ^i^Department of Human Development and Quantitative Methodology, University of Maryland, College Park, MD 20742;; ^j^Department of Human Development, Teachers College, Columbia University, New York, NY 10027

We acknowledge the important points raised by the authors of the letter (1) written in response to our article (2). The histories of discrimination, exclusionary policies, and racism in the United States are interwoven contexts with the experience of poverty, and cash alone will not be enough to address all of these sources of inequality (3). We are grateful to the families and communities in this study, and we strive, and will continue to work toward, centering their voices and experiences. Indeed, we hope that the research from this study will spark discussions and ongoing research acknowledging that children are not born into equal circumstances in the United States.

With respect to the empirical implications raised in the letter (1), the Baby's First Years study was not designed with the statistical power to detect small to moderate differences in poverty-reducing effects by race/ethnicity (4). We did not preregister hypotheses regarding differences in impacts by race/ethnicity, immigrant-origin status, child gender, or other indicators of diversity in our sample. However, we will, and we encourage others to, explore how the data arising from this study can be applied in ways to probe and investigate how unconditional income can matter in different ways according to family context and circumstances.

We also want to take the opportunity to clarify the distinction between “increased brain development” as posited in the letter (1) and our study’s findings (2). Our study reports greater high-frequency, but not low-frequency, brain activity in the high-cash gift group, particularly in frontal and frontocentral brain regions; this is distinct from and not a reflection of increased, or accelerated, brain development.
